# Artificial Neural Networks (ANNs) and Response Surface Methodology (RSM) Approach for Modelling the Optimization of Chromium (VI) Reduction by Newly Isolated* Acinetobacter radioresistens* Strain NS-MIE from Agricultural Soil

**DOI:** 10.1155/2019/5785387

**Published:** 2019-05-26

**Authors:** Nur Syuhadah Ram Talib, Mohd Izuan Effendi Halmi, Siti Salwa Abd Ghani, Uswatun Hasanah Zaidan, Mohd Yunus Abd Shukor

**Affiliations:** ^1^Department of Land Management, Faculty of Agriculture, Universiti Putra Malaysia, 43400 Serdang, Selangor, Malaysia; ^2^Department of Agricultural Technology, Faculty of Agriculture, Universiti Putra Malaysia, 43400 Serdang, Selangor, Malaysia; ^3^Department of Biochemistry, Faculty of Biotechnology and Biomolecular Sciences, Universiti Putra Malaysia, 43400 Serdang, Selangor, Malaysia

## Abstract

Numerous technologies and approaches have been used in the past few decades to remove hexavalent chromium (Cr[VI]) in wastewater and the environment. However, these conventional technologies are not economical and efficient in removing Cr(VI) at a very low concentration (1-100 ppm). As an alternative, the utilization of bioremediation techniques which uses the potential of microorganisms could represent an effective technique for the detoxification of Cr(VI). In this study, we reported a newly isolated bacterium identified as* Acinetobacter radioresistens* sp. NS-MIE from Malaysian agricultural soil. The chromate reduction potential of strain NS-MIE was optimized using RSM and ANN techniques. The optimum condition predicted by RSM for the bacterium to reduce hexavalent chromium occurred at pH 6, 10 g/L ppm of nutrient broth (NB) concentration and 100 ppm of chromate concentration while the optimum condition predicted by ANN is at pH 6 and 10 g/L of NB concentration and of 60 ppm of chromate concentration with chromate reduction (%) of 75.13 % and 96.27 %, respectively. The analysis by the ANN model shows better prediction data with a higher R^2^ value of 0.9991 and smaller average absolute deviation (AAD) and root mean square error (RMSE) of 0.33 % and 0.302 %, respectively. Validation analysis showed the predicted values by RSM and ANN were close to the validation values, whereas the ANN showed the lowest deviation, 2.57%, compared to the RSM. This finding suggests that the ANN showed a better prediction and fitting ability compared to the RSM for the nonlinear regression analysis. Based on this study,* A. radioresistens* sp. NS-MIE exhibits strong potential characteristics as a candidate for the bioremediation of hexavalent chromium in the environment.

## 1. Introduction

Chromium is a type of heavy metal that is nowadays ubiquitously present in water, soil, and air. Heavy metals allude to metallic elements which have reasonably high density and possess harmful and toxic effects even at a very low concentration [1, 2]. Chromium was initially founded in 1797 by a scientist named Louis Nicolas Vauguelin as a component in the red crystalline mineral crocoite (PbCrO_4_). It was first described as a shiny grey metal that is unscented, hard crystalline, tasteless, and lustrous. Chromium is one of the elements in the transition group VIB besides tungsten and molybdenum in the twenty-fourth position of the periodic table [3]. Nearly 0.037% of the earth's crust is comprised of chromium making it as the seventh most abundant element in the earth. It is significantly more abundant than molybdenum, copper, lead, cobalt, zinc, and cadmium [4]. Chromium exists in several oxidation states ranging from +2 to +6 with the most stable being the trivalent and hexavalent chromium forms. Both trivalent and hexavalent chromium possess different biological effects. Trivalent chromium is a naturally occurring chromium and it is not harmful to living organisms while hexavalent chromium is carcinogenic and mobile as it can be transported through the cells via the sulphate and phosphate routes [5].

Throughout the last decades, hexavalent chromium has grown to be one of the major pollutants that contribute to the ecosystem's imbalance and substantial environmental problem due to its potential carcinogenicity and toxicity to human beings and living organisms [6]. This situation may arise as a result of rapid industrialization, especially in developing countries. Due to its lustrous and hardly corrosive characteristics, hexavalent chromium is used widely in various industries such as electroplating, inks, wood preservatives, textile dyeing, leather tanning, pigment production, refractories, and metal refining [7]. The high concentration of hexavalent chromium discharges as a result from industry is threatening the public health and quality of potable water. Therefore, the concentration of hexavalent chromium must be reduced and minimized to an acceptable and safe level. According to the US Environmental Protection Agency (EPA), the maximum contaminant level issued for total chromium is 100 *μ*g L^−1^. For hexavalent chromium, the maximum concentration issued by the World Health Organization is 50 *μ*g L^−1^ [6].

Numerous technologies and approaches have been studied and developed in the past to remove hexavalent chromium such as ion exchange, chemical precipitation, adsorption, and biosorption, electrodialysis, and reverse osmosis [8, 9]. These technologies have been practiced for decades due to their efficacy. However, several disadvantages are arising from the utilization of these conservative approaches such as not economical and expensive to be operated in the long run, high energy requirements, and production of toxic discharge along with secondary effluent. Furthermore, some of them are not suitable and have low capacity to remediate low concentrations of hexavalent chromium [10]. Thereby, bioremediation has been introduced as one of the alternatives to remove heavy metals including hexavalent chromium.

Bioremediation is a biological process that remediates the environment through processes like adsorption, redox transformation, and precipitation reactions. Microorganisms such as bacteria, fungi, microalgae, and actinomycetes play a very vital and important role in reducing hexavalent chromium in bioremediation of industrial wastewater and contaminated soil. The reduction of hexavalent chromium by bacteria occurs aerobically, anaerobically, or both, relying on the factors affecting the reducing efficiency and the microbial species itself. Hexavalent chromium reducing aerobes usually employ NADH and endogenous cell reserves as their mechanism to reduce hexavalent chromium and hexavalent chromate-reducing anaerobes using the electron transport system containing cytochromes to reduce hexavalent chromium [11]. This biological technique of using microorganisms to reduce hexavalent chromium is a very fast and cost-effective process. Moreover, the utilization of microorganisms offers the reduction of hexavalent chromium at a very low concentration of chromium [12]. A large variety of bacteria have been reported for their ability in reducing and transforming hexavalent chromium to trivalent chromium under aerobic and anaerobic conditions such as* Intrasporangium* sp. Q5-1,* Bacillus* sp. ES29,* Escherichia coli*,* Enterobacter cloacae,* and* Pseudomonas fluorescens* LB300 [13].

It was proven that bioremediation by bacteria is one of the promising methods to remove heavy metals from wastewater or soil. The process is often affected by many parameters depending on the type of heavy metals, sample nature, and bacteria used. Therefore, the optimization process during bioremediation is one of the essential steps to achieve the best result. Response surface methodology (RSM) and artificial neural network (ANNs) are the examples of optimization tools that can be used to improve the bioremediation process. The conventional one factor at a time (OFAT) optimization methods that have been practiced since ages ago not only is laborious and time-consuming, but also does not show the overall interactions and effect of each parameter tested during the experiment and it may provide inaccurate data [14, 15].

RSM is one of the statistical and mathematical methods developed to overcome the issue as it can be used to measure the effects of numerous independent variables and the response of the experiment. It is beneficial to determine the effects of each variable alone or in combination [15]. This method serves many advantages such as the fact that it is time effective, is inexpensive, and provides an accurate and precise result. It is practical in the present study which is to optimize the Cr(VI) reduction by a newly isolated bacteria. This approach implements the low-order polynomial equation in a predetermined region of the variables and the equation will be assessed to obtain the optimum values of the variables tested for the best responses [14, 16, 17]. Besides RSM, artificial neural network (ANN) has also been broadly researched to optimize process because of its effective, robust, and prominent features in capturing the nonlinear relationships between parameters and response in a complex system. This method is very practical to be applied especially in a process requiring complex mechanisms to function, which is the case with the biological treatment process for heavy metals pollution. Thus, the employment of ANNs has gathered a growing interest in wastewater treatment control and modelling [14, 18]. Quite an increasing number of researchers around the globe have started to employ RSM and ANN as tools to predict and optimize metal removal processes like the removal of Cu(II) by alkali-modified spent tea leaves [19] and the removal of Pb(II) by the nanocomposites of rice straw [20]. The results and data from the implementation of RSM and ANN were superior in their sensitivity analysis, generalization capabilities, and predictive efficiency in the bioremediation of heavy metals from wastewater and environment compared to OFAT alone [14, 15].

This study was carried out to isolate, characterize, and optimize chromate reduction from several chromate-reducing bacteria from different agricultural soil samples. The availability of efficient hexavalent chromium reducing microorganisms is a crucial requirement for future bioremediation of agricultural soils contaminated with hexavalent chromium. It was discovered that most chromate-reducing bacteria were isolated from nonagricultural sources such as from industrial effluents and mining. Therefore, the isolation of a new potent strain is significant. The effect of pH, type of media, media concentration, and hexavalent chromate concentration on chromate reduction by strain NS-MIE were optimized and modelled using two different approaches, RSM and ANN. Hopefully, the findings will provide a broad understanding of newly isolated chromate-reducing bacteria and provide alternative bacteria to bioremediate the hexavalent chromium in soils and water.

## 2. Materials and Methods

### 2.1. Isolation and Screening of Chromate-Reducing Bacteria

The chromate-reducing bacteria were isolated from two different soil sources which were agricultural soil and industrial soil. The bacteria from the agricultural soil were isolated from an oil palm plantation situated in Universiti Putra Malaysia (2.988208” N, 101.730392” E) and bacteria from industrial sources were isolated from the Juru Industrial Park, Pulau Pinang (5.316293” N, 100.430008” E). Soil from both sources were collected randomly and placed in a sterile Falcon tube. Then, 5 g of the soil was diluted in 100 mL of nutrient broth and incubated for 48 h on a 170 rpm rotary shaker at 28°C. After incubation, the broth was diluted using the serial dilution method and spread on nutrient agar supplemented with 20 ppm, 50 ppm, and 100 ppm of K_2_Cr_2_O_7_ and incubated for 48 h at 28°C. Next, a loop of the primary culture on the spread plate was streaked on nutrient agar plate supplemented with 50 ppm of K_2_Cr_2_O_7_. The plates were incubated at 28°C for 24 h and continually streaked until pure colonies were obtained. Then, 1% of the original culture was aerobically grown overnight. 0.1 mL of the grown culture was inoculated in a 10 mL medium containing 50 ppm of K_2_Cr_2_O_7_. The mixtures were then incubated on a rotary shaker (170 rpm) at 28°C. After incubation, 1 mL of the sample was withdrawn aseptically and centrifuged at 10, 000 × g for 10 min. The supernatant of the sample was used to measure the chromate reduction rate using the 1,5-diphenyl carbazide method. A total of seven bacteria with the highest chromate reduction rate were further screened in a media supplemented with 100 ppm of chromate.

### 2.2. Molecular Identification of Chromate-Reducing Bacteria

Pure bacteria colony exhibiting the highest chromate reduction was further identified using molecular identification method. 16s rRNA gene of the chosen isolate was amplified by PCR using universal forward primer and reverse primer. Then, it was analyzed for amplification using 1.2% agarose followed by electrophoresis at 70 V for one h. The amplified 16s rRNA gene fragment was purified and sent to Apical Scientific Sdn. Bhd. for sequencing. The obtained 16s rRNA gene sequences were exported into “Basic Local Alignment Search Tool” (BLAST) from the website of the National Centre for Biotechnology Information (NCBI) to identify the highest match. The output of the BLAST sequences was sorted based on highly similar identity with other genus or species in the GenBank records. Next, the phylogenetic tree was constructed using MEGA software version 10.0 [21].

### 2.3. Screening of Chromate-Reducing Media

The bacterium chosen from the previous screening was further screened using different types of media. The media used in the screening were nutrient broth, Luria Bertani, and minimal salts media. The bacteria were incubated in each media that were initially supplemented with 50 ppm of K_2_Cr_2_O_7_ for 48 h on a 170 rpm rotary shaker at 28°C. All media used were autoclaved at 121°C, 15 psi for 20 min before being used. The reduction rate was measured using the 1,5-diphenyl carbazide method at 24, 48, 72, 96, 120, and 124 h after exposure.

### 2.4. Assaying Chromate Reduction by 1,5-Diphenylcarbazide

The activity of chromium reducing bacteria was measured by colorimetric changes using 1,5-diphenyl carbazide (Sigma, USA). The colorimetric reagent was prepared by dissolving 0.025 g in 100 mL of analytical grade acetone in order to minimize deterioration. Next, the hexavalent chromium in a grown culture was assayed by mixing 400 *μ*l of the culture supernatant with 400 *μ*l and 200 *μ*l of the colorimetric reagent in a 1mL cuvette. The mixture was then further analyzed spectrophotometrically at 540 nm [22]

### 2.5. Optimization of Chromate Reduction by Response Surface Methodology (RSM)

The optimization of the chromate reduction was carried out using response surface methodology (RSM). Three independent variables which were pH, media concentration, and chromate concentration were chosen as the parameters with the reduction of chromate as a response of the experiment. All of the parameters were studied based on the range as stated in Table 1. The Box Behnken design was selected as the design to run all 17 optimization experiments.

The experiment was conducted in triplicate based on Table 2 with chromate reduction rate as the response of the experiment. Innoculation of 10 mL of the 1% original culture from 3, 6.5, and 10 g/L of the nutrient broth containing 50, 75, and 100 ppm of hexavalent chromium at different initial pHs were incubated for 24 h on a 170 rpm rotary shaker at 28°C. Tris and potassium phosphate buffers were used to regulate the pH of the media. After incubation, 1 mL of the sample was withdrawn aseptically and centrifuged at 10000 ×g for 10 min. The supernatant of the sample was used to measure the chromate reduction rate using the 1,5-diphenyl carbazide method [15].

### 2.6. Optimization of Chromate Reduction by Artificial Neural Network (ANN)

The optimization of the chromium reducing activity by ANN was conducted using a commercial ANN software, NeuralPower version 2.5 (CPC-X Software). The optimization was trained and tested by different types of the learning algorithm, which were backpropagation, Levenberg–Marquardt, and conjugate gradient methods before multilayer feed and multilayer full feedforward approaches were carried out. The network architecture consisted of an input layer with four neurons, an output layer with one neuron, and a hidden layer. Chromium concentration, pH, and media concentration were used as network inputs and chromate reduction rate was used as the output. One hidden layer, number of neurons layers, and the transfer functions of hidden and output layers were used to develop several networks and to determine the optimal network topology. Each layer of the network was trained until the network root of mean square error (RMSE), average correlation coefficient (R), and average determination coefficient were lower than 0.01 and equal to 1 and 1, respectively [19].

### 2.7. Determination of Optimum Point Using RSM and ANN

The optimum point for chromate reduction by strain NS-MIE was determined and identified using RSM and ANN. In RSM, the approach involved the desires and priorities for every variable to figure out the relationship between chromium reduction rate and each of the variables involved [15]. Numerical optimization function in the Design Expert software recommended four optimum points with high desirability of the chromium reduction rate. In ANN, the optimum points were determined by comparing three different algorithms which were particle swarm optimization, rotation inherit optimization, and genetic algorithm. Determination of the optimum conditions by each of the algorithm was carried out using NeuralPower version 2.5 (CPC-X Software). The parameters used were pH, nutrient broth concentration, and chromate concentration. The predicted optimum condition by RSM and ANN was further validated, and the deviation from the optimum point was determined.

### 2.8. Comparative Error Analysis of RSM and ANN Models

Error analyses such as root mean square error (RMSE), correlation coefficients (R^2^), standard error of prediction (SEP), and relative percent deviation (RPD) were calculated between experimental and predicted data of both RSM and ANN models. The formula used for error analysis was

(1) R2=1−∑i=1nYi.p−Yi.e2∑i=1nYi.p−Ye2RMSE=∑i=1nYi.p−Yi.e2nSEP=RMSEYe×100RPD=100n∑i=1nYi.e−Yi.pYi.e

where *Y*_*i*.*e*_ is the experimental data, *Y*_*i*.*p*_ is the predicted data, *Y*_*e*_ is the mean value of experimental data, and n is the number of the experimental data. The modelling ability of a given model is depending on the RMSE and SEP value as the lower the RMSE and SEP value, the higher the modelling ability. To evaluate the modelling abilities of the RSM and ANN models, the values predicted by RSM and ANN models were plotted against the corresponding experimental values.

### 2.9. Effect of Different Initial Concentration of Hexavalent Chromium on Chromate Reduction Rate and Bacterial Growth

About 1% of the bacterial culture was aerobically grown overnight. Then, several 10 mL medium supplemented with different concentration of K_2_Cr_2_O_7_ (50, 60, 70, 80, 90, 100, 110, 120, and 160 ppm) were prepared in universal bottles. Exactly 0.1 mL of the grown culture was inoculated in each bottle containing the medium at the different initial concentration of K_2_Cr_2_O_7_. They were then incubated on a 170 rpm rotary shaker at 28°C. At every 2 h for the first 24 h, 1 mL of the sample was centrifuged at 10,000 ×g and the supernatant was assayed and measured at 540 nm. The growth of the bacterium was measured by diluting the pellet with distilled water and measured at 600 nm. After the 24th h, the reading was taken every 6 h of the experiment until the 96th h. Media without inoculant but supplemented with same different initial concentration were used as negative control of the experiment.

## 3. Results and Discussion

### 3.1. Isolation, Identification, and Screening of Chromium Reducing Bacteria

**Figure 1:**
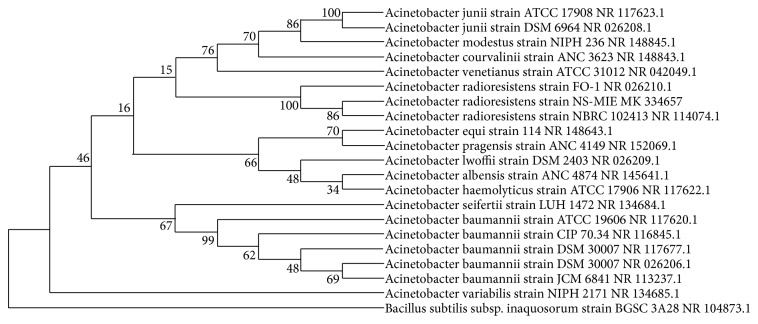
Phylogenic tree of* A. radioresistens* strain NS-MIE (Accesssion no: MK334657). The 16s rRNAs of* A. radioresistens* strain NS-MIE and* Acinetobacter radioresistens* strain NRBC 1024013 NR shows a high bootstrap value of 86 %.

Based on the biochemical analysis, the best chromate-reducing bacterium was a Gram-negative and aerobic bacterium. The bacterium needs oxygen to continuously grow and to fulfill its role to catalyze the hexavalent chromium bioreduction. The bacterium was identified as* Acinetobacter radioresistens* strain NS-MIE (Accession no. MK334657) by 16s bacterial sequencing. The phylogenetic analysis reveals that the NS-MIE strain originates from the* A. radioresistens* family through several genomic variations along with various generations with a high bootstrap value of 86% (Figure 1). None of the related strains from the phylogenetic analysis shows chromium resistant characteristic. This could happen on the account of genomic variations that took place along numerous genomic variation and modifications. The bacterium* A. radioresistens* sp. NS-MIE must have maintained its novel characteristics of chromium resistance and tolerance. The bacterium was chosen because it has the highest amount of chromium reduction rate in the screening process.

The broth containing soil from two different sources which were agricultural soil and industrial soil were spread on agar plates supplemented with 20, 50, and 100 ppm of K_2_Cr_2_O_7_. The bacterial count from each plate decreases with an increasing level of chromium causing sensitive microorganisms to cease growth on chromate-supplemented agar plates. This phenomenon was also reported by Das et al. [23]. From the agar plates integrated with chromate, a total of 40 bacteria of different colonies and morphologies were then further isolated in pure form and were subjected to assessment for relative chromate resistance.

For the primary screening, a total of 40 pure colonies were screened in nutrient broth incorporated with 50 ppm of K_2_Cr_2_O_7_. The chromate reduction rate of all 40 colonies was measured spectrophotometrically after 48 h of incubation. From the result, it was found that only 17.5% of the tested bacteria were resistant and able to reduce more than 70 % of 50 ppm K_2_Cr_2_O_7_ after 48 h of incubation. The tolerance of these 7 selected colonies were then screened secondarily in 100 ppm of K_2_Cr_2_O_7_ to evaluate their ability to reduce chromium in high concentration and short duration. Based on the performance of these bacteria in reducing 100 ppm of chromate, bacterium SS1, SS6, and SS21 were discovered to be appealing and promising because they reduce more than 90 % of the chromate after 72 h of incubation (Table 3). Considering the rate of chromate reduction and the duration of the bacterium to reduce the chromate, isolate SS1 was chosen in this study.

### 3.2. Screening of Chromate-Reducing Media

Growth medium or culture medium is significant to assist the growth and sustainability of microorganisms. There are several types of media designed to be suitable for growing different types of microorganism. The types of the most common culture media used are nutrient broth, Luria Bertani, and minimal salt media. Each of these different media provides the nutrients needed for the microorganisms to sustain themselves, survive, and grow. They contain many ingredients enabling the media to be a favor to different types of microorganisms. Glycerol and glucose are frequently used as carbon sources for the microbes while nitrates and ammonium salts are employed as inorganic nitrogen sources of the media [24].

*A. radioresistens* sp. NS-MIE was grown in different types of media including nutrient broth, Luria Bertani, and minimal salt media to identify the types of media that is the best and most suitable for the bacterium to reduce hexavalent chromium. Within 48 h of the incubation period, bacteria* A. radioresistens* sp. NS-MIE was able to reduce up to 94.21 % and 83.41 % of hexavalent chromium in nutrient broth and Luria Bertani, respectively (Table 4). These findings can be supported by many recent studies that used nutrient broth and Luria Bertani under an aerobic condition as the medium for the chromate-reducing medium [2, 25, 26]. Luria Bertani is a complex media and the utilization of Luria Bertani as a medium in chromate reduction is less favorable in some research. Besides that, the employment of minimal salt media for chromate-reducing bacteria was found to be effective but it requires a longer incubation period in order to reduce hexavalent chromium completely [27]. Considering all the significant aspects, the nutrient broth was chosen as the chromate-reducing media in this study.

### 3.3. Optimization of Chromate Reduction by Response Surface Methodology (RSM)

Response surface methodology is a statistical tool that explains the relationship between multiple independent variables and one or more responses. It has been extensively used as a method to design experiments. This RSM technique is dependent on the fit of mathematical models like linear, quadratic models square polynomial function, and others [28]. In RSM experiments, the verification of the models was attained by means of statistical methods. This method can be utilized specifically to enhance the efficiency of the optimization experiment. The fundamental concept of the experimental design by RSM is to branch out all the vital considerations and merge the results via a mathematical model. The model can be employed for interpretations, predictions, and, most importantly, optimization. The optimization process by RSM is separated into six steps which are the assortment of the independent variables and responses, choosing the experimental design, carrying out the experiments and collection of data, fitting the model equation, analysis of variance, and determination of the optimal point [29].

In this study, optimization by RSM was carried out to procure a quadratic model of the bioreduction of the hexavalent chromium. The independent variables chosen for the study were NB concentration, pH, and chromium concentration with chromium reduction rate as the responses of the experiment. The experimental and predicted results are represented in Table 6. The predicted value of the experiment was obtained from an equation. The equation below has been derived from several regressions analyses to clarify the chromium reducing activity by bacteria* A. radioresistens* sp. NS-MIE.

(2) Y=69.95+16.26A−14.48B−13.62C−2.52AB+0.44AC+1.36BC−8.42A2−1.62B2−3.52C2

Y in the equation refers to the predicted chromium reduction rate while A, B, and C are coded parameters for NB concentration, pH, and chromium concentration, respectively. The ANOVA analysis was executed to evaluate the crucial effect of each of the single variables and combined variables (Table 5). Based on the ANOVA analysis, it is notable that the single variable of A (NB concentration), B (pH), and C (chromium concentration) was statistically significant with p < 0.0001. The combined variables of A2 were also statistically significant. The chromate reduction increases as the NB concentration increases, pH decreases, and chromium concentration decreases. The media containing 75 ppm of chromium, 10 g/L NB, and pH 6 can enhance the chromate reduction up to 95.49 %. The regression model was significant with p < 0.05 and the R^2^ value of 0.9974. The adjusted R^2^ value of the experiment is 0.9888, implying that there is only a 2 % variation of the chromium reduction activity by* A. radioresistens* that cannot be explained by this model. Moreover, the good correlation between the experimental and predicted value of the chromium reduction rate was also observed based on the adjusted R^2^ value that was close to 1.

### 3.4. Optimization of Chromate Reduction by Artificial Neural Network (ANN)

Artificial neural network (ANN) is a type of linear modelling techniques that has been widely used to explain a wide range of processes and mathematical objects. ANN procedures include the selection of a network architecture, determination of hidden layers and number of neurons in each layer, learning, training, and, lastly, validation and verification of the data [30]. In this study, ANN was utilized to model the chromium reduction rate by multilayer feedforward neural networks using QuickPropagation as the learning algorithm to determine the weight and biases [15]. Multilayer feedforward is a type of network that is commonly used and known in ANN modelling. These networks consist of three other types of layers which are input layer (independent variables), several hidden layers, and output layer (dependent variables). The inputs applied in this study were NB concentration, pH, and chromium concentration while the output was chromium reduction rate. The number of the hidden layers between inputs and output has to be overcome by overfitting and overtraining to build a better ANN model. The result achieved from the experiment (Table 6) was used to build the model.

**Figure 2:**
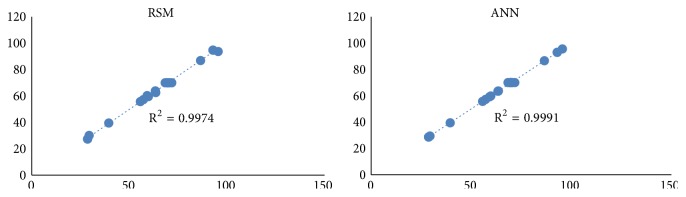
Actual versus RSM and ANN predicted values for chromium reduction by* A. radioresistens* strain NS-MIE.

The experimental result of the chromium reduction by* A. radioresistens* sp. NS-MIE by RSM studied earlier was used randomly for training and testing. Based on Table 6, the bold form is the data used as a testing dataset (Table 7) for ANN modelling and the remaining data were used as a learning dataset (Table 8). Table 6 also displays the ANN predicted values for each experiment. The results of the ANN predicted values show a close correlation between the actual and the predicted values. This implies that the ANN result can fit the actual experimental data precisely. The relationship between actual experimental data and ANN predicted values can be viewed by plotting the experimental data versus ANN predicted. The value between actual and predicted ANN is close, signifying that the nonlinear fitting effects of the model are good. R^2^ and RSME values of the experiment were used to evaluate the accuracy of the model. The R^2^ value is 0.9998 which is very close to 1, indicating that this model gives a good prediction.  Figure 2 shows the comparisons between RSM and ANN experimental versus predicted value. From the graph plot, ANN shows better fitting with higher R^2^ value which also implies that ANN gives better optimization result compared to RSM study. To estimate and predict the responses, multiple layers of network and topologies were used to identify the exact number of neurons in the hidden layer between inputs and output layers. The ideal five ANN models are represented in Table 9.

### 3.5. Determination of Optimum Point Using RSM and ANN

The optimum points of* A. radioresistens* sp. NS-MIE to reduce hexavalent chromium were determined using RSM and ANN. This method uses priorities and desires to measure the relationship of every parameter involved with the chromium reduction rate as the response of the experiment. The optimum points predicted by RSM were determined by numerical optimization in the Design Expert software 6.01. The method displays the desirability value of 0.886 for the maximum chromate reduction rate by* A. radioresistens* sp. NS-MIE. The maximum chromate reduction of 75.13 % was achieved at the optimized condition of 10 g/L of nutrient broth, pH 6, and 100 ppm of chromium concentration.

The prediction of the optimum point by ANN was done by three different algorithms which were genetic algorithm, rotation inherit optimization, and particle swarm optimization. The result shown in Table 10 reveals that there is no significant difference in values of chromium reduction rate predicted by the three different algorithms. The optimum point predicted by both RSM and ANN shows close agreement between the experimental and the predicted values implying that the obtained model is adequate to optimize the chromium reduction by* A. radioresistens* sp. NS-MIE. Both RSM and ANN can be concluded as the appropriate model to use for prediction and optimization process.

### 3.6. 3D Dimensional Analysis

**Figure 3:**
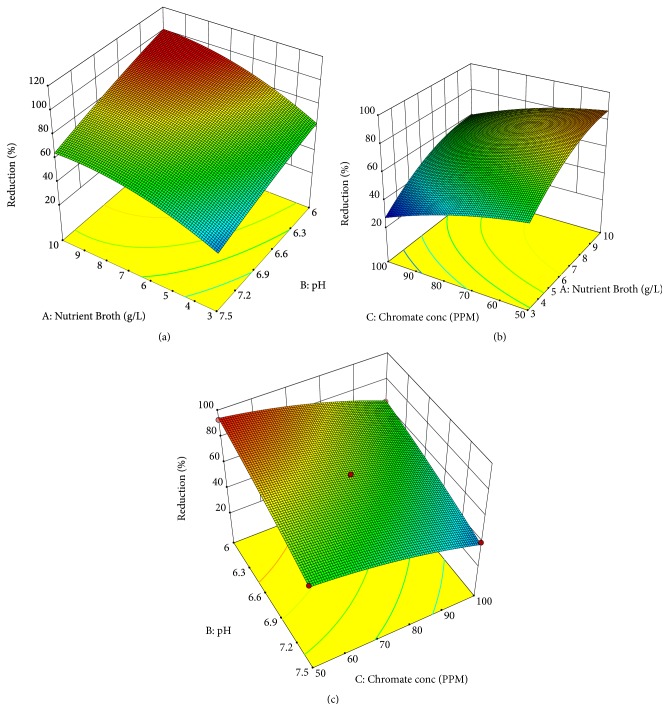
Three-dimensional surface curve on the interaction between three independent variables by RSM. Surface interaction curve between, (a) NB concentration versus pH, (b) NB concentration versus chromium concentration, and (c) pH versus chromium concentration.

**Figure 4:**
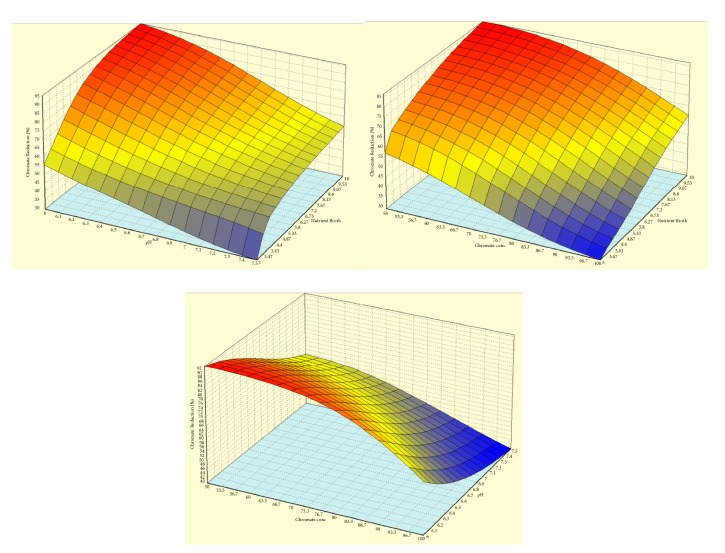
ANN response surface for three different independent variables. Response surface between NB concentration versus pH, NB concentration versus chromium concentration, and pH versus chromium concentration.

Figures 3 and 4 show the response surface plot showing the effect of interactions between the three variables on chromate reduction by* A. radioresistens* sp. NS-MIE by RSM and ANN. The 3D contour plots represent the interactive effect of two variables at one time by keeping the value of the other parameter. The interaction involved in the study was the interaction between nutrient broth concentration with pH, nutrient broth concentration with chromium concentration, and chromium concentration with pH. The elliptical shape of the contour displays that there is an interaction between those particular parameters. When there is no interaction between the parameters, the 3d contour plot shows a circular or round shape.

Both the RSM and ANN response surface plots show similar patterns of interaction between each of the parameters. The interaction between pH and NB concentration shows that chromium reduction rate was increased with an increasing amount of NB concentration and pH value of the medium. According to a study by Fan et al. [31], low pH is favorable in hexavalent chromium reducing activities because it aids in the redox reaction of the aqueous phase and it helps the proton to participate in the following reaction:

(3) $$\begin{array}{c} {HCrO}_{4}=+7H^{+}+3e\;4\;{Cr3}^{+}+4H2O \end{array}$$

The interaction between NB concentration and chromium concentration shows that, with the increasing amount of chromium concentration and decreasing amount of NB concentration, the chromium removal rate seems to be increasing. From the plot, it can be seen that NB concentration in the range from 8 to 10 g/L brought chromium reduction rate to a stable increasing pattern. The interaction between pH and chromium concentration displays a declining chromium reduction rate with the increasing pH values and chromium concentration. This is explained earlier by the interaction between NB concentration and pH where low pH is stated to be more preferable due to the redox reaction.

### 3.7. Comparative Error Analysis of RSM and ANN Models

Table 11 represents the comparative error analysis of the RSM and ANN models. The root mean square error (RMSE), correlation coefficients (R^2^), standard error of prediction (SEP), and relative percent deviation (RPD) were obtained from evaluating the experimental and predicted values of both models. The comparative error analysis was made to verify the prediction accuracy and generalization capacity of both models in optimizing the Cr(VI) removal by* A. radioresistens.* The correlation coefficients (R^2^) for the RSM and ANN models were 0.9974 and 0.991, respectively, indicating that the ANN model shows better regression and fitting compared to RSM (Figure 2). The high R^2^ value also reveals that only ± 0.1% of the data cannot be explained by the models of both RSM and ANN. The RSME values in Table 9 indicate the absolute fit of the model. The standard error prediction (SEP), also known as sums of square error, and the relative percent deviation (RPD) show greater value for RSM as compared to ANN. This indicates that RSM model prediction has a greater deviation in comparison to the prediction made by the ANN model. It also means that the ANN model provides better fitting and higher accuracy in predicting the percentage of Cr(VI) reduction by* A. radioresistens*. Some studies made in the past that used to compare the utilization of RSM and ANN approaches in removing heavy metals such as the removal of lead from leachate using red mud [32] and removal of copper by alkali-modified spent tea leaves [19] also reported that ANN is a better approach as an optimization tool.

### 3.8. Effect of Hexavalent Chromium Concentration on the Percentage of Chromate Reduction and Bacterial Growth

**Figure 5:**
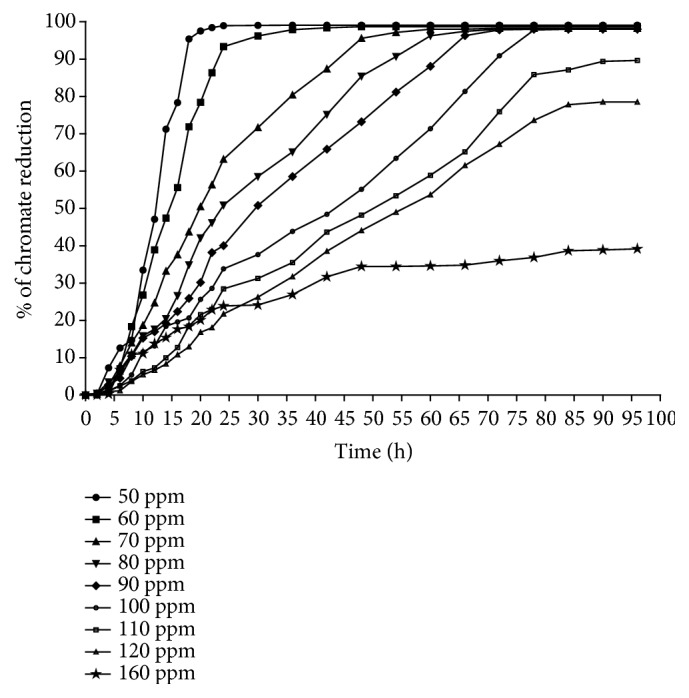
Effect of different initial concentration of hexavalent chromium on chromate reduction rate. The percentage of chromate reduction seems to decrease as the concentration of the hexavalent chromium increase. The chromate reduction rate seems to remain stagnant at certain value starting from the hexavalent chromium concentration of 110 ppm and above.

The effect of chromium concentration on the percentage of chromate reduction and bacterial growth is significant in the study because it implies the tolerance level of the bacteria to chromium. The bacterium was grown aerobically in NB media supplemented with nine different initial concentrations of the chromium (50 to 160 ppm). In this study, the bacterium* A. radioresistens* strain NS-MIE has the ability to reduce 90 % to 99 % of 50 and 60 ppm hexavalent chromium within the first 24 h (Figure 5). Within the next 48 h, the bacterium reduces 95 % and 85 % of 70 ppm and 80 ppm of hexavalent chromium, respectively. Finally, 90 and 100 ppm of hexavalent chromium were reduced completely in the next 78 h of the experiment.* A. radioresistens* strain NS-MIE can reduce up to 39 % of the 160 ppm chromium. However, the chromate-reducing activity seems to be stagnant without any significant increase after 48 h of the incubation period. The bacterium is incapable of reducing the hexavalent chromium completely starting from the hexavalent concentration of 110 ppm and above. The percentage of chromate reduction is stagnant after a certain amount of incubation time, which might be due to the inhibition of chromate reductase in* A. radioresistens*, thereby affecting the capability of the bacterium to reduce Cr(VI) at the higher concentration [33].

**Figure 6:**
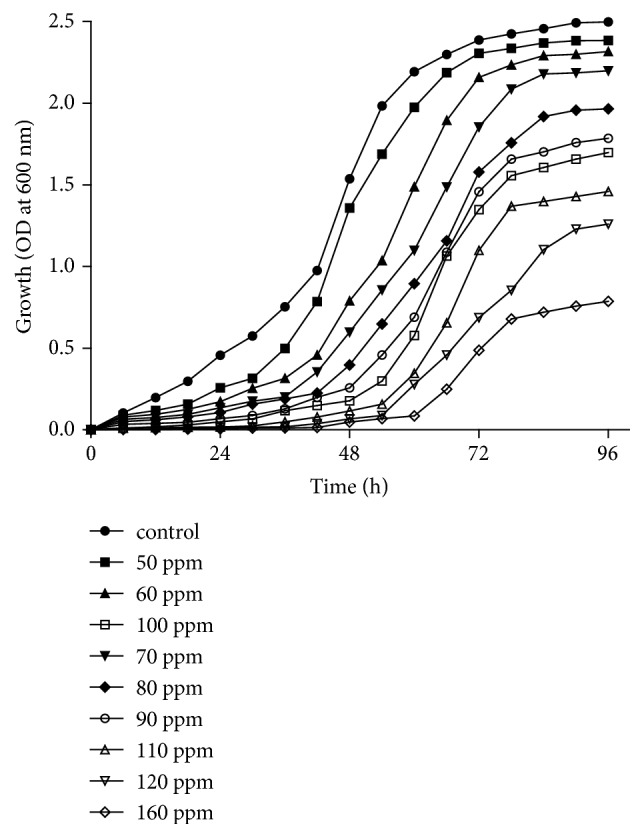
Optical density of* A. radioresistens* strain NS-MIE at different concentrations of hexavalent chromium.

The effect of chromium concentration was further evaluated by measuring bacterial growth every 6 h from the initial point of the experiment until the growth reaches a stationary phase. From the experiment, it was observed that the bacterial growth keeps decreasing as the hexavalent chromium concentration increases (Figure 6). In the media containing 50 and 60 ppm of hexavalent chromium,* A. radioresistens* strain NS-MIE's growth continuously rose with a growth rate that was double its initial point at the first 48 h of incubation. At 160 ppm, the bacterium grew very slowly and finally reached the stationary phase after 78 h of incubation. Most studies regarding the bacterial kinetics in the presence of Cr(VI) show fair increasing values of bacteria growth during the initial period of the experiment. This might be because microorganisms usually develop resistance to certain types of heavy metals and they are metabolically adapting to the presence of heavy metals in their surroundings during acclimatization [34]. Some studies reported that the metal ion damaged the existing enzyme in the microorganisms and the enzyme will be replaced with a new metabolically adapted enzyme [35, 36]. However, the effect of the acclimatization might not be seen after a certain amount of time. This happens because, in the presence of elevated Cr(VI), growth is inhibited resulting in a decreased amount of bacterial growth [34].

## 4. Conclusions

The newly isolated bacterium* A. radioresistens* sp. NS-MIE shows and offers strong potential in reducing hexavalent chromium even at a high concentration of hexavalent chromium. The bacterium exhibits high tolerance and resistance in NB supplemented with 160 ppm of K_2_Cr_2_O_7_ but only reduces hexavalent chromium up to 94.75 % under the optimum conditions. The optimization of chromium reduction by the bacterium* A. radioresistens* sp. NS-MIE by RSM and ANN was successfully executed, and it was found that optimization by ANN gives better estimation point and data fitting as compared to RSM. Thus, it is envisioned that the bacterium provides a promising potential to be utilized in bioremediation of chromium-contaminated sites and the biological treatment of wastewater.

## Figures and Tables

**Table 1:** Upper limit and lower limit of Box Behnken Design.

Variables	Unit	Range and level		
		-1	0	+1
pH	-	3	6.5	10
Media concentration	g/L	7.5	6.75	6
Chromate concentration	ppm	50	75	100

**Table 2:** Design of Box Behnken.

Run	Nutrient Broth Concentration [g/L]	pH	Chromate concentration [ppm]
1	6.5	7.50	50
2	10	6.00	75
3	6.5	6.75	75
4	6.5	6.00	50
5	10	6.75	100
6	3	6.00	75
7	6.5	6.75	75
8	6.5	6.00	100
9	10	6.75	50
10	3	7.50	75
11	6.5	6.75	75
12	3	6.75	50
13	6.5	6.75	75
14	6.5	7.50	100
15	5.5	6.75	75
16	3	6.75	100
17	10	7.50	75

**Table 3:** Percentage of chromate reduction of bacteria incubated in media supplemented with 100 ppm of K_2_Cr_2_O_7_.

Bacterium	Percentage of chromate reduction [%]		
	24th h	48th h	72nd h
SS1	78.45	97.21	98.01
SS6	72.56	81.23	92.33
SS17	60.23	75.35	83.28
SS21	74.21	85.25	92.43
SS28	67.32	73.81	82.18
SS34	66.12	70.23	79.98
SS35	71.28	81.32	88.12

*∗*h: hour

**Table 4:** Screening of chromate-reducing media.

Media	Chromate reduction (%)
Nutrient Broth	94.21
Luria Bertani	83.41

*Minimal salt media*	
Fructose	9.42
Galactose	12.53
Glucose	16.11
Maltose	7.56
Sucrose	10.14
Glycerol	7.98
Mannitol	7.11
Starch	13.27
Sodium acetate	12.87

**Table 5:** Analysis of variance (ANOVA).

Sources	Sum of squares	df	Mean squares	F-value	P>F	
Model	5697.40	9	633.04	158.45	< 0.0001	*significant*
A-Nutrient broth	2116.31	1	2116.31	529.69	< 0.0001	
B-pH	1677.11	1	1677.11	419.77	< 0.0001	
C-Chromate conc.	1483.92	1	1483.92	371.41	< 0.0001	
AB	25.42	1	25.42	6.36	0.0397	
AC	0.76	1	0.76	0.19	0.6755	
BC	7.40	1	7.40	1.85	0.2158	
A^2^	298.19	1	298.19	74.63	< 0.0001	
B^2^	11.06	1	11.06	2.77	0.1401	
C^2^	52.07	1	52.07	13.03	0.0086*∗*	
Residual	27.97	7	4.00	4.61		
Lack of Fit	21.69	3	7.23		0.0869	*Not significant*
Pure Error	6.27	4	1.57			
					R^2^	0.9974
					Adjusted R^2^	0.9888

*∗*P > F less than 0.05 = statistically significant.

**Table 6:** Central composite design matrix for the three independent variables with the observed and predicted response for chromium reduction by *A. radioresistens* sp. NS-MIE.

Run	NB conc. [g/l]	pH	Cr[VI] conc. [ppm]	Cr[VI] reduction [%]		
				Observe response	RSM predicted	ANN predicted
1	6.5	7.50	50	63.5663	62.60	63.566
2	10	6.00	75	95.4913	93.18	95.491
3	6.5	6.75	75	71.7557	69.95	69.952
**4**	6.5	6.00	50	92.8957	94.27	92.896
5	10	6.75	100	59.7617	61.10	59.762
6	3	6.00	75	55.6473	55.61	55.647
7	6.5	6.75	75	68.3594	69.95	69.952
**8**	6.5	6.00	100	63.3438	64.31	63.344
9	10	6.75	50	86.5347	87.47	86.535
10	3	7.50	75	29.3827	31.69	29.383
**11**	6.5	6.75	75	69.4251	69.95	69.952
12	3	6.75	50	57.1515	55.81	57.151
**13**	6.5	6.75	75	70.3893	69.95	69.952
14	6.5	7.50	100	39.4539	38.08	39.454
15	6.5	6.75	75	69.8326	69.95	69.952
**16**	3	6.75	100	28.6329	27.70	28.633
17	10	7.50	75	59.1438	59.18	59.144

**Table 7:** Testing dataset for ANN modelling.

Run	NB conc. (g/L)	pH	Cr(VI) conc. (ppm)	Cr(VI) reduction (%)
4	6.5	6.00	50	92.8957
8	6.5	6.00	100	63.3438
11	6.5	6.75	75	69.4251
13	6.5	6.75	75	70.3893
16	3.0	6.75	100	28.6329

**Table 8:** Learning data set for ANN modelling.

Run	NB conc. (g/L)	pH	Cr(VI) conc. (ppm)	Cr(VI) reduction (%)
1	6.5	7.5	50	63.5663
2	10	6.0	75	95.4913
3	6.5	6.75	75	71.7557
5	10	6.75	100	59.7617
6	3	6.0	75	55.6473
7	6.5	6.75	75	68.3594
9	10	6.75	50	86.5347
10	3	7.5	75	29.3827
12	3	6.75	50	57.1515
14	6.5	7.5	100	39.4539
15	6.5	6.75	75	69.8326
17	10	7.5	75	59.1438

**Table 9:** Summary of active networks.

Model	Learning algorithm	Connection type	Transfer function output	Transfer function hidden	Training set R2	RSME	Testing set R2	RMSE
3-20-1	QP	MNFF	Tanh	Tanh	0.99945	0.626	0.99985	0.345
3-20-1	QP	MNFF	Tanh	Sigmoid	0.99345	0.826	0.92985	0.245
3-20-1	QP	MNFF	Tanh	Linear	0.99145	0.926	0.91985	0.545

**Table 10:** Validation of the optimization values predicted by RSM and ANN.

Model	Algorithm	NB conc [g/L]	pH	Chromate conc. [ppm]	Predicted RSM/ANN [%]
RSM	Desirability function	10.0	6.0	100.0	75.13

ANN	Genetic algorithm	10.0	6.0	60.17	96.26
	Rotation inherit optimization	10.0	6.0	60.18	96.26
	Particle swarm optimization	10.0	6.0	61.19	96.25

**Table 11:** Comparative error analysis of RSM and ANN models.

Error		Model	
		RSM	ANN
Root mean square error	RMSE	0.6781	0.302
Correlation coefficients	R^2^	0.9974	0.9991
Standard error of prediction	SEP (%)	2.1900	0.3300
Relative percent deviation	RPD (%)	1.9984	2.5700

## Data Availability

The data used to support the findings of this study are available from the corresponding author upon request.
